# Bisphenol A, Chapter 2: New Data Shed Light on Exposure, Potential Bioaccumulation

**DOI:** 10.1289/ehp.117-a210b

**Published:** 2009-05

**Authors:** Tanya Tillett

Bisphenol A (BPA), an industrial chemical used in a variety of consumer products, is ubiquitous in the modern environment, with residues found in the urine of an estimated 93% of Americans over 6 years of age, according to data from the 2003–2004 National Health and Nutrition Examination Survey (NHANES). Recent research indicates that BPA acts as an endocrine disruptor and may increase the risk of heart disease, diabetes, and liver problems in adults. Until now, most exposure was thought to occur through diet, and the chemical was thought to clear the body quickly and completely. But a new study shows that urine BPA levels of subjects who had fasted for several hours were not as low as expected, suggesting either nondietary exposures or accumulation in fatty tissue, or both ***[EHP 117:784–789; Stahlhut et al.]***.

Although BPA is fat-soluble and thus can accumulate in fatty tissues, animal and human data suggest it tends to be rapidly metabolized, with elimination thought to be virtually complete within 24 hours of acute exposure. To gain a better understanding of how BPA clears the body, investigators in the current study used data from 1,469 adult participants in the 2003–2004 NHANES. Study participants (excluding children and insulin-dependent diabetics) had been asked to fast for at least 6–9 hours. Using the urine drawn from each study participant, the investigators modeled log BPA concentration against fasting time, adjusting for urine creatinine and other confounders, to estimate what they called the “population-based half-life” of BPA for a 0- to 24-hour fasting period.

Previous studies have reported that BPA has a urinary elimination half-life of only 4–5 hours, but BPA levels in this population declined much more slowly, showing a drop from adjusted population peak to trough levels of only 46% by 17 hours. Although there was a relatively rapid decline in BPA levels during the 4.5- to 8.5-hour fasting interval, the BPA slope was essentially flat between 8.5 and 24 hours, suggesting very slow or minimal elimination during that time.

The findings are consistent with two possible explanations—first, that BPA exposure occurs through means other than food, and second, that BPA accumulates in body fat, from which it is gradually released over time. The authors conclude that their findings highlight the need for additional research on chronic BPA exposure, identification of significant nonfood sources of exposure (which may include dental composites and sealants, household dusts, air, recycled and carbonless paper, and the PVC pipe approved for use in residential water supply lines in many cities), and confirmation of reported data on bio-accumulation of the xenoestrogen in human adipose tissue. Confirmation of the current findings could lead to a reevaluation of BPA exposures in risk assessment studies.

## Figures and Tables

**Figure:**
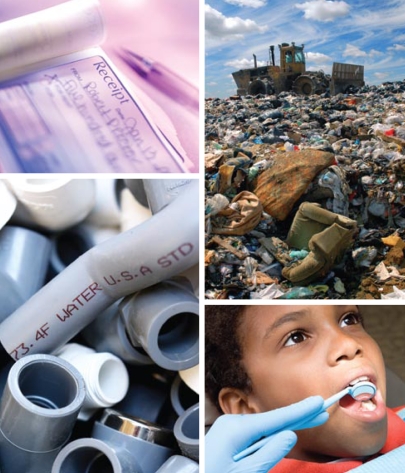
New findings raise the possibility that nonfood BPA exposure may be more substantive than previously thought.

